# Paradigm change to cost-effective predictive and preventive medicine: individual profiling of healthy vasospastic individuals for targeted prevention as the cost-effective personalised medicine

**DOI:** 10.1186/1878-5085-5-S1-A155

**Published:** 2014-02-11

**Authors:** Olga Golubnitschaja, Kristina Yeghiazaryan, Josef Flammer

**Affiliations:** 1 Department of Radiology, Rheinische Friedrich-Wilhelms-University of Bonn, Germany; 2 Department of Ophthalmology, University Hospital Basel, Switzerland

## Paradigm change to cost-effective predictive and preventive medicine

A paradigm change from delayed interventional approaches to cost-effective predictive and preventive medicine presents an innovative concept for advanced healthcare. Early detection of individual predispositions in healthy populations followed by preventive treatments tailored to the person should be committed to the obligations of primary healthcare.

## Vasospastic Syndrome: definition and symptoms

Vascular deregulation (VD) or Vasospastic Syndrome is defined as an inappropriate constriction or insufficient dilatation in the microcirculation. Primary VD is highly prevalent in subpopulations of young people and can potentially predispose healthy individuals to severe disorders (neurodegeneration and cancer) being, therefore, particularly attractive for predictive diagnostics and primary prevention in targeted sub-populations [1], namely

**Figure 1:**
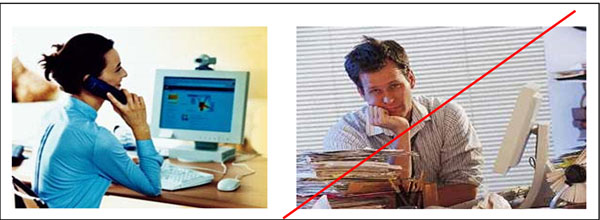
Symptoms and typical attitude of individuals with Vasospastic Syndrome: individuals with primary vasospastic deregulation tend to a meticulous personality and successful professional career such as the young female on the left image [2]

- it occurs more frequently in females and is manifested at puberty, moderating with age

- this phenomenon is even more frequent in the Japanese population compared to Caucasians

- usually, academics are more affected by VD

- in addition to clinical signs there is an inborn increased sensitivity to any kind of stress provocation (mechanical, cold, emotion, etc.), altered drug sensitivity, frequently cold extremities, altered sleep behaviour, reduced feeling of thirst but increased smell perception, low blood-pressure, reduced body-mass-index, more frequent migraine compared to general population

- Compared to the general population, vasospastic individuals tend to have a meticulous personality and successful professional career, Figure 1.

## Patient-profiling to diagnose individual predisposition before pathology manifestation

Clinical observations, sub-cellular imaging and “gene hunting”-investigations provide evidence for vasospasm as a predisposition to glaucoma [3], the second leading cause of blindness in human beings worldwide. Irrespective of glaucoma predisposition, a development of further vasospasm-related pathologies in individuals with (untreated) vasospastic syndrome cannot be excluded. Patient-profiling and predictive blood-tests detecting pathology-specific biomarker-patterns can specify individual predisposition for effective prevention at low cost.

## Patient-profiling for positive and negative prediction of pathology development

**Figure 2:**
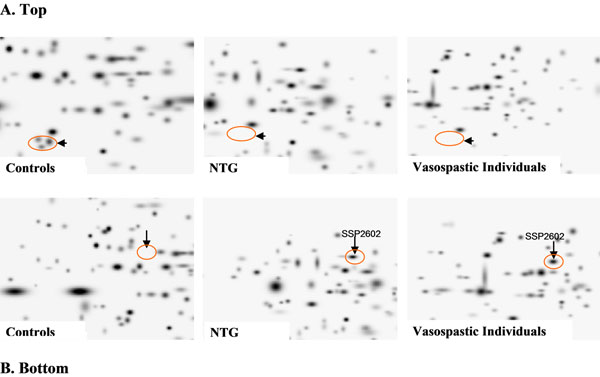
Proteomics-imaging of blood-biomarkers (*ex vivo* identification in circulating leukocytes) specific for normal-tension glaucoma (NTG). **A. Top:** The pathology-specific protein-cluster is completely suppressed in both NTG and vasospasm in contrast to controls. **B. Bottom:** The marked protein SSP2602 is highly up-regulated in both NTG and vasospasm; this protein normally is not expressed by circulating leukocytes of controls – healthy individuals without vasospasm [4]

Whereas positive prediction for patients at high risk is important to recognize the pathology before it manifests and at early stages, the negative prediction for individuals at low risk help to avoid undesirable treatments and invasive approaches in the case of non-predisposed individuals. Individual patient profiles are utilised for patient modeling and risk assessment. Blood tests utilizing “omics”-technologies are useful for creating accurate non-invasive positive/negative predictive approaches (Figure 2) [4].

## Conclusions and expert recommendations

- The expression similarities between glaucoma and VD *versus* controls indicate, on one hand, a predisposition of VD individuals to glaucomatous damage, and, on the other hand, an important role of a vascular component in glaucoma pathology.

- Expression dissimilarities between VD and glaucoma patients might indicate some glaucoma-specific pathomechanisms that are not involved in the stage of predisposition by VD.

- Both expression similarities and dissimilarities could be useful in ascertaining the diagnosis of glaucoma, positive and negative prediction.

- Molecular rearrangement in leukocytes of both VD and glaucoma patients has been shown to be typical for circulating leukocytes during vascular injury and includes an up-regulated adhesive protein expression via ICAM1, an induced chemo-taxis via P2Y purinoceptors, a mobilisation of intracellular Ca^2+^ response via Na^+^/Ca^2+^ exchanger, and the core of tissue remodelling metalloproteinases.

- Pathology-specific molecular and sub-cellular patterns may create the basis for the development of more specific and non-invasive molecular imaging technologies in early/predictive glaucoma diagnostics.

- Development of some other “down-stream” pathologies different from glaucoma but related to primary vasospastic syndrome cannot be excluded and should be investigated through well designed large-scaled studies.

- Predictive molecular-profiling in blood can specify individual predisposition for effective prevention to low costs [5].
